# Lifestyle predictors of successful aging: A 20-year prospective HUNT study

**DOI:** 10.1371/journal.pone.0219200

**Published:** 2019-07-11

**Authors:** Ingunn Bosnes, Hans Morten Nordahl, Eystein Stordal, Ole Bosnes, Tor Åge Myklebust, Ove Almkvist

**Affiliations:** 1 Clinic for Mental Health and Substance Abuse, Nord-Trøndelag Hospital Trust, Namsos, Norway; 2 Department of Psychology, Norwegian University of Science and Technology (NTNU), Trondheim, Norway; 3 Department of Mental Health, Norwegian University of Science and Technology (NTNU), Trondheim, Norway; 4 Division of Psychiatry, St. Olavs Hospital, Nidaros DPS, Trondheim, Norway; 5 Department of Research and Innovation, Møre and Romsdal Hospital Trust, Ålesund, Norway; 6 Department of Psychology, Stockholm University, Stockholm, Sweden; 7 Department of Neurobiology Care Sciences and Society, Karolinska Institutet, Stockholm, Sweden; Nathan S Kline Institute, UNITED STATES

## Abstract

**Background:**

Lifestyle factors predicting successful aging as a unified concept or as separate components of successful aging are important for understanding healthy aging, interventions and preventions. The main objective was to investigate the effect of midlife predictors on subsequent successful aging 20 years later.

**Materials and methods:**

Data were from a population-based health survey, the Nord-Trøndelag Health Study (HUNT), with an average follow-up of 22.6 years. Individuals free of major disease at baseline in 1984–86 with complete datasets for the successful aging components in HUNT3 in 2006–08, were included (n = 4497; mean age at baseline 52.7, range 45–59, years). Successful aging was defined either as a unified category or as three components: being free of nine specified diseases and depression, having no physical or cognitive impairment, and being actively engaged with life. The midlife predictors (smoking, physical activity, alcohol consumption, obesity and social support) were analysed both as separate predictors and combined into a lifestyle index controlling for sociodemographic variables, using multivariable regression analysis.

**Results:**

Successful aging as a unified concept was related to all the lifestyle factors in the unadjusted analyses, and all except alcohol consumption in the adjusted analyses. The individual components of successful aging were differently associated with the lifestyle factors; engagement with life was less associated with the lifestyle factors. Non- smoking and good social support were the most powerful predictors for successful aging as a unified concept. When the lifestyle factors were summed into a lifestyle index, there was a trend for more positive lifestyle to be related to higher odds for successful aging.

**Conclusions:**

Lifestyle factors predicted an overall measure of SA, as well as the individual components, more than 20 years later. Modifiable risk factors in midlife, exemplified by social support, may be used for interventions to promote overall health and specific aspects of health in aging.

## Introduction

It is now well established that we live longer than previous generations. Although longevity may be a goal in itself, the quality of life in old age may be just as, or even more important, than the number of years added to the lifespan, for both individuals and society in general. No formal definition of successful aging (SA) exists, but there is general acceptance that SA should include freedom from chronic diseases and good physical and mental functioning [1, 2]. A recent study found that older people’s plans and wishes for successful aging related to activities, engagement with life, and health [3]. It is apparent that SA is a multicomponent concept; Rowe and Kahn’s three-component model contains three elements: absence of disease and disability, high cognitive and physical functioning, and engagement with life [1, 4]. Because aging is a lifetime process, an individual may be aging successfully at one point in their lives but not at others [5] or with respect to one component of SA but not another; thus, the heterogeneity of SA in older persons should be investigated [2, 6].

However, there are other alternative conceptualizations of SA as exemplified by other research [7]. The Rowe and Kahn model has in particular been criticized for being a biomedical model with too little emphasis on psychosocial factors, individuals’ subjective meanings of SA, and that the focus on individual responsibility for health may overshadow the importance of structural factors [7–9]. In this study, SA has been studied as a unified concept and as its separate components, which includes social factors as its third component. The present study is a development of a previous study investigating the prevalence of successful aging and its correlates [10].

The objective for the Rowe and Kahn model of SA has been to identify early and midlife predictors of later usual or successful aging for prevention purposes. The lifestyle risk factors smoking, physical inactivity, alcohol consumption, obesity, and poor diet have been consistently linked with single negative health outcomes like chronic disease, disability or premature mortality [11–13]. Social support has also been linked with mortality [14], population health [11], and cognitive and physical performance [15, 16]. These findings indicate that age-related chronic diseases and mortality are highly associated with several modifiable factors present earlier in the life course [12, 17], but these risk factors’ relative contribution to the outcomes, especially midlife factors, are less studied and understood [12]. Furthermore, information about the relationships between these factors and SA as a unified concept including absence of disease, high functioning and engagement with life and its different components are much sparser [12, 18, 19]. In addition, studies with prospective longitudinal designs investigating the relationships between risk factors in midlife and SA many years later are rare, despite their importance, but those published do indicate that risk factors in midlife are associated with SA [12, 18–21]. Lastly, several studies of SA have large numbers of participants, but few are population-based, as required to obtain valid epidemiological data.

The aim of this longitudinal study was to investigate prospectively the relationships between the specific lifestyle factors (physical activity, smoking, alcohol consumption, obesity and social support) in midlife and a multidimensional concept of SA more than 20 years later in a population-based sample.

## Materials and methods

### Ethics statement

All three HUNT studies are based on informed written consent. HUNT1 was approved by the Norwegian Data Protection Authority and HUNT3 was approved by the Norwegian Data Protection Authority and by the Regional Committee for Medical and Health Research Ethics. At the time of HUNT1, the regional committees was not yet established. This study was separately approved by the Regional Committee for Medical and Health Research Ethics (REC no 2013/1116).

### Study population

The Nord-Trøndelag Health Study (HUNT) is a large population-based epidemiological health survey, which to date has been conducted four times in Nord-Trøndelag County in Norway (HUNT1 1984–86, HUNT2 1995–97, HUNT3 2006–08, and HUNT4 2017–19). All inhabitants in the county aged 20 years or older have been invited to attend [22] and the response rates were high for HUNT1 (89.4%) and acceptable for HUNT3 (54.1%). Self-reported lifestyle information was drawn from HUNT1 for this study, and there was an average follow-up time of 22 years before the outcomes were measured in HUNT3.

The target population of this study was aged 45–59 years at baseline (HUNT1, 1984–86) and 70–89 years, with complete datasets for the successful aging variables, at follow-up (HUNT3, 2006–08). A total of 144 participants were excluded because of reporting present or earlier presence of myocardial infarction, angina pectoris, diabetes, or stroke at baseline. Respondents younger than 70 years at follow-up were also excluded because they were given a different questionnaire in the HUNT3 study. Of 6314 possible participants, 1664 were excluded because of missing values for the outcome variables in HUNT3. Those with missing values for the outcome variables were more likely to be older (p<0.001) and have had fewer than nine years of education (p<0.001).

### Lifestyle factors

We included the following lifestyle factors: physical activity, smoking, alcohol consumption, obesity and social support; the relative risk ratio was calculated for each of these. Lifestyle factors were explored both as single factors and as a lifestyle index. Smoking, physical activity, alcohol consumption and social support were assessed from questionnaires 1 and 2 in HUNT1, while obesity was assessed from the medical examination data.

#### Smoking

Smoking status was characterised as current smoker, former smoker or never smoked.

#### Alcohol consumption

Alcohol consumption was assessed from the answers to two questions: “How often did you drink alcohol (beer, wine or spirits) during the last 14 days?” and “Have there been periods in your life when you drank excessively or too much?” Low consumption was defined as drinking alcohol fewer than five times in the past 14 days and never having had periods of drinking excessively or too much. Moderate/high consumption was defined as drinking alcohol five times or more in the past 14 days or answering “yes” or “not sure/maybe” to having had periods of drinking excessively or too much.

#### Physical activity

Physical activity was characterised by the frequency and intensity of activity. Low activity was defined as exercising less often than once a week or exercising once a week at low intensity (no sweating or being out of breath). High activity was defined as exercising once a week or more and, on at least one of these occasions per week, exercising at moderate or high intensity (sweating/out of breath or exhausted).

#### Obesity

Obesity was defined as a body mass index (BMI) ≥ 30.

#### Social support

Social support was characterised by the answers to two questions: “Do you often feel lonely?” and “If you became ill and were bedridden for an extended period of time, how likely is it that you would receive the necessary help and support from family, friends or neighbours?” The compound variable for social support was (1) low, if the respondent answered that they often/sometimes felt lonely and were uncertain/unlikely/highly unlikely to receive the necessary help, (2) medium, if the respondent felt lonely often/sometimes or was uncertain/unlikely/highly unlikely to receive the necessary help, and (3) good, if the respondent rarely/never felt lonely and felt rather/extremely likely to receive the necessary help.

As in previous research, three sociodemographic variables were included as covariates: age, gender, and educational level (split into three levels), see Table 1.

**Table 1:** Baseline characteristics of the study population in the HUNT1 study (1984–86).

Baseline characteristics		Sample (N (%))
Age groups		
	45–49	1238 (27.5)
	50–59	3259 (72.5)
Gender		
	Female	2447 (54.4)
	Male	2050 (45.6)
Education		
	<9 years	2433 (54.1)
	9–12 years	1119 (24.9)
	13 or more years	400 (8.9)
	Missing data	545 (12.1)
Smoking status		
	Never smoked	1857 (41.3)
	Former smoker	1134 (25.2)
	Current smoker	1042 (23.2)
	Missing data	464 (10.3)
Physical activity		
	Active	1901 (42.3)
	Inactive	2090 (46.5)
	Missing data	506 (11.2)
Alcohol consumption		
	Low consumption	3624 (80.6)
	Medium/high consumption	423 (9.4)
	Missing data	450 (10.0)
Obesity		
	BMI <30	4055 (90.2)
	BMI ≥30	419 (9.3)
	Missing data	23 (0.5)
Social support		
	Poor social support	328 (7.3)
	Medium social support	1124 (25.0)
	Good social support	2586 (57.5)
	Missing data	459 (10.2)

### Successful aging definition

SA was defined in line with Rowe and Kahn’s three-component concept [1, 23–25]: (i) absence of disease, including absence of depressive symptoms to assess psychological aspects; (ii) high physical and cognitive functioning; and (iii) active engagement with life. SA was measured on a continuum, to ensure that different levels of SA were considered.

SA was assessed using self-reported health information from The HUNT3 Survey (2006–08). For details regarding the procedures, see Bosnes et al. [10]. The overall SA variable for each participant was obtained from the number of outcomes that satisfied the SA criteria. There were four possible outcomes: SA in no components, SA in one component, SA in two components, or SA in all three components.

#### Absence of disease

The first SA component was defined as the lack of a self-reported history or presence of any of the following diseases: myocardial infarction, angina pectoris, heart failure, other heart disease, stroke/ brain hemorrhage, chronic bronchitis, emphysema or chronic obstructive pulmonary disease (COPD), diabetes, or cancer. All questions had to be answered with no to be classified as absence of disease. In addition, absence of depression was defined by the depression subscale of the Hospital Anxiety and Depression Scale (HADS-D) with a HADS-D score <8 [26, 27].

#### High cognitive and physical functioning

High physical functioning was defined as being able to perform the following activities independently: walk around indoors on the same floor, go to the toilet, wash themselves, take a bath or shower, dress and undress, go to bed and get up, eat, prepare warm meals, do light house-work (e.g.: wash dishes), do heavier house work (e.g.: wash floors), do the laundry, do the shopping, pay bills, take medicines, go out, and take the bus. Respondents reporting an inability to perform one or more of these activities independently were recorded as having impaired physical functioning. High cognitive functioning was defined as never having trouble remembering what happened some days ago. If they reported having trouble with this “sometimes” or “often”, the respondents were classified as having impaired cognitive functioning. All the questions on functioning and memory had to be answered at follow-up for a person to be included in the study.

#### Active engagement with life

Respondents were classified as being actively engaged with life if (1) they were currently in paid or unpaid work or (2) they had gone to a museum/art exhibition, a concert, the theatre, a film, church/chapel, or a sports event or had participated in community service, a choir, theatre work or church work at least once a month over the last six months. Respondents were included if they answered at least one of the questions in HUNT3. All questions had to be answered negatively to be classified as non-active at follow-up.

### Statistical analysis

Statistical analyses were performed with STATA version 15.0 software [28]. Multivariable ordinal logistic regression techniques were used. The ordinal regression model evaluated the associations with increasing levels of SA. Firstly, we assessed the relationships between single lifestyle factors and SA. Secondly, the lifestyle factors (smoking, physical activity, alcohol consumption, obesity and social support) was summed to create the lifestyle index (number of favourable predictors: 1–5) and the combined effect of this on SA was examined. Thirdly, we investigated the effect of the lifestyle factors on each of the three components of SA. Results are presented as odds ratios (ORs) with 95% confidence intervals (95% CI). The level of significance was set to p <0.05.

## Results

The study included 4497 participants, with a mean age at baseline of 52.7 years ±3.6 years and at follow-up of 75.3 ±3.5 years; 54.4% were women, see Table 1. For baseline characteristics regarding the lifestyle factors, see Table 1. Of the participants, 15.6% met all three SA criteria at HUNT3, while a large proportion met one (34.4%) or two (35.5%) criteria, and 14.5% met none of the criteria. Those who met all SA criteria, were more likely to be women (p<0.001), younger (p<0.001), and to have a higher level of education (p<0.001) compared to those meeting no SA criteria, see Table 2.

**Table 2:** Comparison of baseline characteristics between participants with different levels of successful aging.

Baseline characteristics	SA in no criterion	SA in one criterion	SA in two criteria	SA in all criteria
**Sociodemographic factors**				
Age, years	53.4±3.6	53.0±3.5	52.5±3.6	51.9±3.4
Married (%)	88.8	88.4	88.8	88.6
13+ years of education (%)	6.1	8.9	8.9	11.5
Gender, female (%)	43.6	49.1	57.4	69.6
**Lifestyle factors**				
Non- smokers (%)	58.0	63.3	69.7	74.1
Physically active (%)	39.5	45.8	49.9	54.2
BMI <30 (%)	87.6	89.6	90.9	92.2
Good social support (%)	58.9	63.0	66.0	66.8
Low alcohol consumption (%)	85.3	89.1	90.2	93.0

The adjusted associations between the lifestyle factors and levels of SA and the three components of SA are provided in Table 3. There was no interaction between lifestyle factors and gender, so both genders were combined in the analyses. All lifestyle factors were significantly associated with SA as a unified concept in the unadjusted models, and all except alcohol consumption remained associated in the adjusted models. Based on the adjusted analysis, the odds of SA were significantly increased for both former smokers and those who had never smoked, compared to current smokers. The odds of SA were also significantly higher for more physically active respondents than for less active respondents, for those with BMI <30 than for those with BMI ≥30 and for those with good social support than for those with poor social support, see Table 3. There was no significant difference between those with medium level versus those with poor social support.

**Table 3:** Likelihood of SA as a unified and split concept (multivariate regression analyses) as adjusted (n = 3769) odds ratios (ORs).

Lifestyle factors		SA in all criteria	Absence of disease (SA1)	High function (SA2)	Active engagement with life (SA3)
		Adjusted ORs (95% CI)^a^	Adjusted ORs (95% CI)^a^	Adjusted ORs (95% CI)^a^	Adjusted ORs (95% CI)^a^
Smoking					
	Current smoker	1	1	1	1
	Former smoker	**1.52 (1.30, 1.79)** ***	1.39 (1.16, 1.66)**	1.25 (1.04, 1.50)*	1.37 (1.14, 1.64)**
	Never smoked	1.74 (1.50, 2.03)***	1.65 (1.40, 1.95)**	1.09 (0.92, 1.30)	1.78 (1.50, 2.11)***
Physical activity					
	Low	1	1	1	1
	Moderate/high	1.25 (1.11, 1.41)***	1.20 (1.05, 1.37)**	1.15 (1.00, 1.31)*	1.18 (1.03, 1.35)*
Alcohol consumption					
	Moderate/high	1	1	1	1
	Low	1.12 (0.92, 1.36)	1.02 (0.81, 1.27)	1.29 (1.03, 1.63)*	0.97 (0.78, 1.22)
Obesity					
	BMI ≥30	1	1	1	1
	BMI <30	1.39 (1.14, 1.71)**	1.54 (1.22, 1.94)***	1.23 (0.98, 1.55)	1.05 (0.84, 1.33)
Social support					
	Poor	1	1	1	1
	Medium	1.22 (0.96, 1.55)	1.32 (1.01, 1.72)*	1.21 (0.92, 1.57)	0.95 (0.73, 1.24)
	Good	1.54 (1.23, 1.93)***	1.52 (1.18, 1.95)**	1.37 (1.06, 1.76)*	1.17 (0.91, 1.50)

^a^ OR: Adjusted for age, gender and educational level

* = p <0.05

** = p <0.01

*** = p <0.001

Numbers in bold types are significant at least at p< 0.05

Table 3 shows that the SA components were somewhat differently related to the risk factors. For SA as a unified category and the component SA1 (absence of disease), the findings were quite similar. Non-smoking and good social support seemed to be the two most important lifestyle factors. For SA2 (high physical and cognitive function), the factors had less impact, but good social support seemed to be the strongest factor. For SA3 (active engagement with life) non-smoking was again the most important lifestyle factor. Overall, non- smoking and moderate/high physical activity were the only two factors related to all criteria and the unified SA concept.

A lifestyle index was created to study the effect of an increasing number of positive lifestyle factors on SA as a unified concept. At baseline, very few participants (1.2%) had none or only one positive lifestyle factor, 8.6% had two, 25.1% had three, 41.8% had four and 23.3% had five. As a result of the small number with no positive lifestyle factors, we combined those with no and one positive lifestyle factor in the analysis. Compared to this group, participants with two or more healthy factors at baseline had greater odds for SA (see Table 4). The benefits of healthy behaviour appeared to increase almost linearly, but the confidence intervals were large.

**Table 4:** Associations between the lifestyle index at baseline and successful aging in HUNT3, as unadjusted (n = 3874) and adjusted (n = 3756) odds ratios (ORs).

Number of positive lifestyle factors^a^	Unadjusted ORs (95% CI)	p	Adjusted ORs (95% CI)^b^	p
1	1		1	
2	2.44 (1.38, 4.29)	0.002	2.06 (1.14, 3.72)	0.017
3	2.51 (1.45, 4.32)	0.001	2.00 (1.13, 3.54)	0.018
4	4.56 (2.65, 7.83)	<0.001	3.52 (2.00, 6.22)	<0.001
5	5.41 (3.13, 9.36)	<0.001	4.27 (2.41, 7.59)	<0.001

^a^ The lifestyle index was a sum of the following positive lifestyle factors: no smoking, high physical activity, low alcohol consumption, no obesity, and good social support

^b^ Adjusted OR: Adjusted for age, gender and educational level

## Discussion

Within this representative sample of adults, there were significant associations between the midlife lifestyle factors non-smoking, higher levels of physical activity, non-obesity and good social support and subsequent SA, 22 years later. Alcohol consumption was not related to SA after adjustment for the sociodemographic variables age, gender and educational attainment, but the other associations remained significant after adjustment. Because the objective of the study was to focus on lifestyle factors, the influence of sociodemographic variables were adjusted for. Therefore, in the following, only lifestyle factors will be discussed.

In the present study, non-smoking and higher physical activity were related to higher odds for SA, but we did not find an association between alcohol consumption and SA. Few studies have investigated the relationships between midlife factors and subsequent SA as a multidimensional concept and its different components. Those that are available have shown links between not smoking at midlife and higher odds of SA [29–31], moderate alcohol consumption and possible (but less certain) higher odds of SA [12, 19, 32], and physical activity [29, 31, 33] or eating fruits and vegetables daily [29, 34] and subsequent SA. The lack of association between alcohol consumption and SA in this study may to some degree be related to the overall low level of alcohol consumption in respondents at baseline, creating low level of variance in alcohol consumption. We tried another definition based solely on frequency of alcohol use, but this did not alter our results. The relationship between alcohol and SA is unclear in other research as well [12, 21]. The relationship between a healthy diet in midlife and subsequent SA could not be investigated in this study, as the HUNT1 study did not collect information on diet, except for the consumption of salt-cured meats or salt-cured fish/herring for dinner.

Social networks may be seen as a structural social support, while perceived social support is perhaps better seen as a functional aspect. These constructs have been noted to be only modestly inter-correlated [14]. In our study, which measured perceived social support, we found that there was no difference between low and moderate levels of social support (those often/sometimes feeling lonely and uncertain/unlikely/highly unlikely to receive the necessary help versus those either often/sometimes feeling lonely or uncertain/unlikely/highly unlikely to receive the necessary help). However, there was a clear difference between the lowest and highest levels of social support (rarely/never feeling lonely and feeling rather/extremely likely to receive the necessary help compared with the lowest level of social support). This suggests that social support is an important predictor of SA in later life. One previous study investigating social networks in midlife and SA found no relationship [29], but a relationship between social networks and health and mortality has been found in other studies [14, 35]. The variability may to some degree be related to the different aspects of social support (social networks and perceived social support).

The lifestyle factors in the present study had to some degree both a general and specific impact on the SA components. Not smoking and higher physical activity had a general impact on the three SA components, while not smoking and good social support seemed to be the two most important lifestyle factors for SA1 (absence of disease) and good social support for SA2 (high physical and cognitive functioning). For SA3 (active engagement with life), non- smoking was again the most important lifestyle factor. In this way, there was a differential pattern of successful aging in relation to the predictors. The results also indicate that good social support may be a more important factor for SA and its components than previously noted [12, 21], as it related to two components of SA and consequently to SA as a unified concept.

In a previous study based on roughly the same sample, high physical and cognitive functioning was the component most difficult to satisfy, closely followed by the component absence of disease [10]. However, a large proportion of the sample (two-thirds) was able to satisfy the criteria for engagement with life. Consequently, it seems possible to be actively engaged with life, regardless of diseases or reduced functional or cognitive ability. The number of positive lifestyle factors included in the lifestyle index was related to the odds for SA. There was an unexpected small drop between two and three lifestyle factors, but overall there was a linear trend for more positive lifestyle factors correlating with higher odds for SA. However, the confidence intervals were quite large and overlapped indicating variability.

In accordance with previous research, the sociodemographic factors younger age [10, 21, 25] and higher educational levels [36, 37] were related to higher odds for SA. Female gender was also related to SA in this study. This may have been because of the SA criteria, since the component absence of disease comprised only major causes of death, such as cancer and heart disease. As heart disease is more frequent among men than women, more women may have been able to satisfy this criterion [10, 24].

Although no formal definition of SA exists, there is some agreement that it should include freedom from major chronic disease, and high functioning. We chose to add active engagement with life to our definition, to be in line with the Rowe and Kahn criteria for SA. The results showed that the SA components absence of disease and high functioning were related to most of the tested midlife predictors, while the component active engagement with life was associated with fewer predictors. SA is therefore clearly not a uniform concept but is complex, measuring different aspects of aging.

Finally, it is worth noticing that at least three lifestyle factors (smoking, physical activity, and alcohol consumption) are related to actions that the individual could master, while social support is related to actions by other individuals. This distinction has implications for possible interventions aiming to influence successful aging.

### Strengths and limitations

One strength of this study is the large population-based sample from an unselected population in a defined geographic area. Another strength is a long follow-up period (22 years). The response rate was also relatively high and a broad range of health-related variables were investigated. Social support as a predictor of SA is also rarely investigated in previous research as is the predictors of the different components.

However, some limitations should be noted. Firstly, as in much epidemiological research, the data in this study were mostly self-reported, with the consequence of uncertainty about the reliability and hence the validity of the results. For instance, the lack of objective cognitive data may have included respondents with cognitive decline in the SA group. Secondly, the definitions and cut-offs of SA and the predictor variables will always to some degree be arbitrary, and this will have influence the results as a function of where the cut-off is set. Thirdly, we acknowledge that aggregation of diseases into one sum score is a simplification and may obscure the relative relevance or weight of the different diseases. In contrast, we can argue that perhaps a multicomponent concept will provide a better description of a lifestyle context of SA, rather than a single or few other factors. Furthermore, we had to exclude respondents with missing SA component values. As noted, those with missing SA component values were more likely to be older and have fewer than nine years of education. This may to some degree have influenced the prevalence estimates. Moreover, other possible important predictor variables for SA, such as genetics, personality and other factors, were not included in this study and we were not able to estimate their role in this study. Lastly, previous analyses from the HUNT study have shown that non-attendance was related to lower socioeconomic status and slightly higher prevalence of chronic diseases [38]. The estimates in this study may therefore be conservative.

## Conclusions

The results of this study have shown that several midlife lifestyle factors were related to subsequent SA as both a unified concept and as different components. The SA components were somewhat differently associated with the lifestyle factors. When the lifestyle factors were summed into a lifestyle index there was a trend indicating that having more than one positive lifestyle factor at midlife resulted in higher odds for SA. The investigated predictors for this SA concept add knowledge about the prerequisites for healthy aging and could be used in health policies and interventions to promote healthy aging.
